# Extra-Limites

**Published:** 1844-07-01

**Authors:** 


### EXTRA-LIMITES.

### On Schools of Naval and Military Surgery. By Sir George Ballingall, M.D.

*To the Editor of the Medico-Chirurgical Review.*

Edinburgh, 10th April, 1844.

Sir,—Some six months ago I addressed a Letter to Sir Robt. Peel, urging the institution of a Professorship of Military Medicine and Surgery in London, and another in Dublin. Thinking that I had thus fully discharged a duty which I owed to the public service, it was not my intention to have entered upon the subject again. But the unfavourable notice of that proposal which has appeared in the last Number of your Journal gives me the opportunity, and suggests the propriety, of offering, to my professional brethren, some explanatory details, which would have been altogether out of place in an address to the Minister.

The first erroneous opinion, which pervades, more or less, the whole review of my letter, is that " my principal object was to contrast the liberal provision made by some Foreign States, particularly Prussia and Austria, for the education of their Army Surgeons with the very scanty provision made for the same purpose by our own Government." This contrast is indeed sufficiently striking, but a little reflection would have shewn that it was only mentioned incidentally, with the view of rousing the attention of Government to the subject; and that my principal object was to point out the difference of duties which devolve on our Military and Naval Surgeons, compared with those of the Continental States. It is the special nature of the duties devolving upon medical officers in our service which constitutes the most important part of the contrast, and which requires a " course of instruction," not " different from," but over and above, " that necessary for the Surgeon destined for practice in civil life." Perhaps nothing will render this contrast more intelligible than the different duties which are expected of a Professor of Military Surgery on the Continent and in this country—there I find numerous gentlemen denominated " Professors of Military Surgery "—one, it may be, teaching the practice of Physic, another perhaps operative Surgery, and a third Ophthalmology; but not one of them, so far as I know, giving a course in the least degree a-kin to mine. If no special instruction be necessary to prepare our Army and Navy Surgeons for their peculiar duties; then are the educational establishments at the general hospitals superfluous. If such special instruction be requisite; then will it be most advantageously given where there are most to profit by it—in the Metropolitan Schools—which are frequented both by the old and the young. To which those already in the service naturally will, and to which those preparing for the service necessarily must resort.

The topics which should be embraced in a course of Military Surgery have of late, been frequently specified, and are pretty fully stated by the reviewer in a quotation from Mr. Marshall—we have here, on the one hand, a number of important topics enumerated as fit to be included in a course of Military Hygiene, and we have, on the other hand, an extended *curriculum,* in no one course of which are these topics embraced. Surely out of twenty months allotted to the practice of Physic, and twenty months to the practice of Surgery—in all forty months, or nearly three years and a half—some six months might be spared to those educating for the Army and Navy, to learn something of the new and peculiar duties which, at their very outset, the emergencies of the service may call upon them to execute. I was not eight-and-forty hours in the Army when I was left in charge of two Battalions, and these receiving recruits every hour.

That the Authorities admit the advantage of such a course of instruction is obvious from their permitting Candidates to take six months Military Surgery in place of six months of the Surgical Attendance enjoined on them. By the regulations of the Army, indeed, the former is preferred, a circumstance which the reviewer has failed to notice. The want of similar sources of instruction elsewhere has long been assigned as a principal reason for not making the course of Military Surgery in this University imperative. Did such a course exist in other Schools I think Government would do well to render attendance on it compulsory, and I do not concur in the opinion that such attendance "might justly be considered a hardship." It is however more for the time than the means that I contend. A prevalent and, I believe, a growing opinion exists, that many of the *curricula*, or courses of education, prescribed by the different licensing bodies, are overloaded with compulsory study. Let there be less *compulsory* and more *optional* study, and I have no fear for the result as regards the Professors of Military Surgery. I have some experience of the popularity of this subject, and although, of late years, for reasons very easily explained, the number of students has been less, I have had, in one year, as many as eighty-one paying pupils, and this without the course being *compulsory* on any one of them.

The study of this department would not be thrown away even upon those "who might not be successful in obtaining appointments in the Army or Navy;"—it would not be thrown away either upon themselves or the public. Let gentlemen who may have so qualified themselves have a preference, in the intermediate approval of recruits, and in those numerous cases where troops are dispersed in small parties over the country, apart from their own Surgeons. The Soldiers would then meet with prompt and efficient treatment, while the public would have always at command a body of men who, without prejudice to their qualifications as general Practitioners, would be conversant with the duties of Military Surgeons, and thus competent to act on every emergency.

In a series of questions, more or less relevant, the reviewer asks, "Is the discipline young officers undergo at Fort Pitt or Haslar, and in a Regiment or on Shipboard, not adapted to initiate them into the mysteries of their respective appointments?" Were I to answer this question, as I shall answer others, in the words of some of the most experienced and intelligent Surgeons in the Army, it would not, I apprehend, suit the reviewer's taste, nor serve his purpose; but I prefer, in this instance, speaking in my own person, and will take the liberty of answering one question, *more Scottico*, by asking another. The promptitude, tact, and efficiency, which an attentive young man ultimately acquires under an intelligent and judicious Surgeon no man can more highly appreciate than I do; but might he not be so prepared, by a course of previous instruction, as to acquire all this more speedily, more surely, and more advantageously to the public service? The instructions given at Chatham and at Haslar I am not disposed to undervalue, but I am not singular in the opinion that much of this instruction might be given with equal, if not with more, advantage elsewhere. The opinion that the Museum attached to the General Hospital at Fort Pitt might be placed more advantageously in the Metropolis, is proved by the fact, that a large subscription, at the head of which is the Director-General, has been entered into by the Medical Officers of the Army, "towards the erection of a building for the Museum and Library in London."

With regard to the general Hospitals, as sources of Clinical instruction, I have already hinted at some of the disadvantages under which they labour, but the subject is by no means exhausted. No general Hospital, in this country, can ever be so advantageously employed as the "Hôpitaux d'Instruction" in France, and for this very obvious reason, that we have not here the same diseases to treat as those with which our Military and Naval Surgeons have to contend in our numerous Colonial possessions. So far as Clinical instruction can be combined with the systematic courses of lectures, which I wish to see established in the Metropolitan Schools, it would perhaps best be given by occasional visits to the several Regimental Hospitals of a large Garrison, like London or Dublin; visits such as I have occasionally made, every Session, to the Hospital in Edinburgh Castle. There the pupils would see the diseases which actually prevail amongst the soldiery in this country—not the diseases of invalids. They would see also the interior œconomy and administration of those Regimental infirmaries which have so long been a source of legitimate pride to the Medical Department of the British Army, and to which no parallel is to be found in the other armies of modern or of ancient times. Few of the subjects embraced in the course of Military Surgery are susceptible of clinical illustration. Many of them are highly susceptible of explanation by means of preparations, casts, models, plans, and drawings, such as are provided for the use of the Students of Military Surgery in this University, and of which I have herewith the pleasure to enclose a printed catalogue. Of the subjects enumerated, at pages 8 and 12 of my Letter to Sir Robert Peel, it so happens that the treatment of Ophthalmia, and of the Venereal Disease, are almost the only subjects admitting of any extensive illustration in the Military Hospitals of Great Britain. The illustration of Tropical Diseases in these Hospitals is obviously impossible; and the soil of England —thanks to our gallant Army and Navy!—has never, in our day at least, afforded a field for clinical instruction in the treatment of Wounds.

General Hospitals, however, are necessary for the purposes of the service. One for the whole Army in Great Britain is certainly not a superfluity, and two for the Navy are perhaps still less so. In the Royal Navy the only establishment akin to the Regimental Hospital is the Sick Berth. This upon most occasions is a very limited field, and to the gentlemen of this Department, when returning from foreign service, or attached to ships lying in port, the great Naval Hospitals, with the Collections and Libraries belonging to them, must prove an interesting and valuable source of professional improvement. But the Gentlemen connected with this branch of the service would not, I think, undervalue the importance of a Metropolitan School, in addition to those means of instruction which are necessarily confined to the great sea-ports. It is only justice to these Gentlemen to say that, amidst all my mixed audience, there are none who have been more punctual in their attendance than some of the Naval Surgeons; there are none from whom I have received more interesting communications; and there are none who have shewn more gratitude for the information I was able to give them in return.

This leads me to say a word upon the attendance of Gentlemen already holding Commissions in the service. I am said to have overlooked the fact, that medical officers, even after many years' absence from their native land, rarely obtain sufficient leave to enable them to study. I had, on the contrary, noticed, to the credit of the present Heads of the Medical Departments of the Army and Navy, that they have granted, for this purpose, every facility which the good of the service would possibly permit. I was myself one of fourteen Army Surgeons who graduated here in one year; I have had in one Session as many as thirty-six medical officers entered to the class of Military Surgery; and in all, during the twenty years I have lectured, four hundred and ninety-eight. This no doubt includes all those gentlemen who have been successively stationed here with their Regiments, several who were upon half-pay, and many who have only attended occasionally, or for very short periods; but, with all these deductions, a very considerable number on leave of absence, many of them in an indifferent state of health, have occupied themselves in a praiseworthy manner, in attendance upon this, and other classes in the University during the whole Session. A numerous body of Medical Officers, those in the service of the Honourable East India Company, have always, from the prolonged leave of absence which they obtain, ample opportunities of attending lectures, and of this they have very fully availed themselves.

I am held to be unfortunate in alluding to the Statistical Reports on the health of the Army and Navy, as sources of information to be turned to account in the

Lecture-room. The conclusions deducible from these reports are indeed "remarkably clear, plain, and unambiguous," and it certainly is not as "the groundwork of a few passing remarks," that the results of these statistical inquiries should be brought before his pupils; but as the most appropriate and most forcible illustrations which a Professor can possibly give, of the observations which it is his duty to make on the geographical distribution of diseases; on medical topography; on the choice of sites for Camps, Cantonments, Barracks, and Hospitals; on the diseases prevalent amongst Europeans on Foreign Stations, and in the vast native Army of India; as well as amongst the various races which constitute our several Colonial Corps, Negroes, Hottentots, Malays, and Cingalese. This, in addition to all other measures for extending their circulation, would be one of the most powerful means of impressing on the rising generation of Army and Navy Surgeons the value of these reports; and I still hold to my opinion that, without this, no justice will be done to them. The measure which I advocate, the establishment of additional Professorships of Military Surgery, has no more strenuous supporter than Major Tulloch, the very author of these reports, and I have reason to believe that this gentleman concurs in every syllable of the following observations by the Editor of the "Naval and Military Gazette."

"No Medical Candidate should be permitted to enter the Army or Navy, who has not attended at least one course of Lectures on Military Surgery, embracing all those circumstances by which the health of Soldiers and Sailors is likely to be affected in different climates; and to enable him to do so, it is indispensable that two medical officers of acknowledged talent in that department of their profession, should be nominated to Chairs in the Universities of London and Dublin, and whose duty it would be, to give to their pupils the benefit of the experience they have acquired in a long course of service, as well as of the vast mass of information annually transmitted, from all foreign stations, to the Medical Departments of the Army and Navy, and which, we feel confident, would most cheerfully be laid open to their inspection for this purpose."

In a subsequent number of the same Gazette, (448,) will be found a communication from several experienced Staff and Regimental Surgeons, who attended the class of Military Surgery in this University during the Session 1840-41. "We rejoice to observe, in a recent number of the Naval and Military Gazette, (January 30th, 1841,) that this subject is taken up in connexion with the valuable statistical inquiries which have lately been made into the sickness and mortality of the Army and Navy, and we are confident that nothing could be more conducive to the public interests than the appointment suggested by the writer, of Professors of Military Surgery in London and Dublin, to whose province it would fall to keep the results of these statistical inquiries constantly before the rising generation of Army and Navy Surgeons."

Amongst the Queries to which I may be expected to reply is the following; "Are the few who have attended the class of Military Surgery, more efficient, more studious, better informed, more accurate observers, or more zealous and industrious in collecting and diffusing the results of their experience and observations, than their brother officers?" This is a somewhat comprehensive question, and it would be somewhat unreasonable to expect me to answer for some seven hundred young men, who have now passed through my hands as pupils, or even for the limited number of these who may have obtained Commissions in the public service. Much of their efficiency must depend upon the "number of hours, which," in the words of the reviewer, "are spent by Medical Officers in the Billiard-room, or dissipated in some of those ingenious methods devised, both in the Army and Navy, to kill time." That, in the opinion of very competent judges, young men so instructed, are well qualified to become efficient medical officers, may be gathered from the following answer to similar questions propounded to two very intelligent and experienced Army Surgeons, Deputy-Inspector Marshall, and the late Staff-surgeon Badenach. "We have a very high opinion of the utility of a course of lectures of this kind. A student who has attended a course of lectures on Military Hygiene, and on Military Medicine, is prepared to avail himself, with great benefit, of the practical advantages of experience, by which means he will be much sooner able to assume in an efficient manner the medical charge of a body of men, than if he had not received such instructions. A course of lectures on Military Surgery is extremely useful, inasmuch as it supplies that information which is peculiarly required by medical officers, and which can be but imperfectly communicated in a course of lectures, either on the practice of Medicine or Surgery."

Again, it is asked, "What practical advantages have resulted, during the last thirty years, from the establishment of a Chair of Military Surgery in Edinburgh?" This is a question very unfit for me to answer, inasmuch as I can scarcely be considered an impartial judge; but, fortunately, I am under no necessity of doing so, the question has been already asked, and already answered, by more competent authority. "Do you think the institution of a class of Military Surgery at Edinburgh, was a beneficial addition to the course of Medical study? In so far as the public service is concerned, the Army, the Navy, and the Service of the Honorable East India Company, I think it has been a most beneficial addition to the Chairs of the Medical School at Edinburgh. Besides Wounds, and what is termed Military Surgery, the Economy of Hospitals, and of Hospital Arrangements, is in this school taught by a gentleman who has served much in the field and in various climates. At the end of the last war upwards of 300 Medical Officers of the Army were placed on half-pay, and it is within my knowledge that many of them profited greatly by attendance upon this class, before they returned to employment on full-pay." Such is the question submitted to, and such is the answer given by Sir James M'Grigor, to the Commissioners for visiting the Scottish Universities in 1827, when I had been about five years in possession of the Chair. With regard to the utility of this class, in subsequent years, I refer the reviewer to a short notice of my Letter to Sir Robert Peel, in the January number of the British & Foreign Med. Review.

Upon one subject, the reviewer's sentiments and mine cordially agree; the propriety of "stimulating the emulative industry of medical officers, and exciting them to professional improvement." Sir Gilbert Blane's Gold Medal as a prize to the Naval Surgeons, is noticed with much approbation in my "Outlines of Military Surgery;" and the successful labours of Mr. Malcolmson, by which he obtained the Prize for the best Essay on "Berriberri and Burning in the Feet," have been quoted in my Class-room from year to year, ever since that Essay was presented to me by the Madras Government. Concurring in all that is proposed upon this point, I am prepared to go even a little further than my reviewer. It is quite possible that a man may write a valuable essay on a given subject, without being an efficient Army Surgeon, and Sir Robert Peel, without taking an additional shilling from the public purse, might offer a boon to industrious students, and to deserving medical officers, ten times more valuable than all the money that can ever be allotted for Prize Essays. For a series of years I have held annually a competition amongst the students of Military Surgery, by way of examination, at the close of the Session, and have been fortunate enough to procure Commissions in the Army or Navy, for several gentlemen who have distinguished themselves in these competitions. No prize can be more valuable, to a candidate for employment in Her Majesty's Service, than a Commission, and none can be more valuable to an officer already in it, than a step of promotion, an appointment to a crack regiment, or to a favourite station. Let this be followed out upon a large scale, let an appointment be given to the most deserving student, and a step of rank to the most deserving officer who may be eligible to it,—their merits, in either case, to be decided by a Committee of experienced officers, as is done at Sandhurst and at Addiscombe. Let this be done annually in each of the three proposed schools, and I can conceive no plan more likely to elevate the character of the

Department, or to furnish the service with a well-informed and efficient body of Staff Officers, capable of giving to the Governor of a Colony, or the Commander of an Expedition, the most judicious advice in all that regards the health of his troops.

I have hitherto, Sir, as you will observe, addressed myself generally to the objections which have been brought in opposition to my views, I would now address myself a little more particularly to you and to the reviewer. I have not presumed to think that these objections embody the sentiments of an experienced Naval Surgeon, more especially of one who has acquired experience on the Indian Station, "who has seen the diversified maladies produced by climate, season, constitution, and co-existing circumstances." I am, indeed, hardly left to conjecture on this subject, for we are told, in a subsequent article, that this reviewer is an accomplished Army Surgeon, of which I make no doubt; but I must be permitted to say, that he has, in my opinion, taken a very limited view of his subject,—a view very far short of those lofty conceptions which dictated the Memoir of Mr. John Bell—a memoir which you have seen proper to reprint, and which, although inapplicable at the present day, as regards the general education of our Army and Navy Surgeons, is *more than ever* applicable to that special instruction which it so forcibly inculcates, and which our enlarged establishments, our extended commerce, and our multiplied possessions demand.

Your reviewer cannot have considered all the important bearings of this question, as regards the *collection* as well as the *diffusion* of information on the health of the troops. The pupils of this class have distinguished themselves in all the recent struggles in India. In the first luckless campaign of Affghanistan, no fewer than three of them perished; one of these was Dr. Cardew, whose fate is so feelingly deplored by Lieutenant Eyre, and who had become so great a favourite with the soldiers that, when unable to travel farther, they endeavoured to save him by conveying his almost lifeless body on the carriage of a gun. To this gentleman I was indebted for a communication on fractures when a pupil of the class, and had his life been spared, I might have been in possession of information which is now for ever lost to the world.

I have at this moment lying before me Statistical tables of the sickness and casualties amongst the Queen's troops in China, and returns of Surgical Cases after Sir Charles Napier's recent actions in Scinde, framed for my special use by the Superintending Surgeon of the force, an old pupil of the class. The communications indeed which I have received from my pupils, in the shape of contributions to the Museum, of Returns and reports from distant stations, or of written Essays on subjects discussed in the progress of the Course, are more than I shall ever live to do justice to, or sufficiently to express my gratitude for. Amongst several of these communications which have appeared in print, I may be allowed to notice an Essay on the Comparative Merits of Flap and Circular Amputations by Mr. M'Hardy, and the Essay on the Feigned Diseases of Soldiers and Seamen, by Dr. Gavin. To these I trust I may soon be enabled to add a valuable history of the Medical Department of the British Army, by Dr. Irving.

I am said to have failed in making out a case for additional Professorships of Military Surgery. Upon this point, however, I trust there is room for a difference of opinion; I am told by a distinguished General Officer, who has fought and bled in the service of his country, that I have made out "a most triumphant case," and we are told by a spirited and intelligent professional writer, "that the Government which reads the arguments of Mr. Guthrie and myself, and does nothing, is as clearly condemned as the man who will persist in denying mathematical axioms."

Will your reviewer now allow me to tell him where I think he has failed? He has failed to see how much the institution of such courses of instruction as I recommend is calculated to raise the character of his department. He has failed to see what influence it is calculated to give to medical opinion in the eyes of those holding superior command; and he has failed to see what a waste of life and of health it may obviate, by a perfect foreknowledge of what has happened, and what may again happen, in any given circumstances.

After having been so freely questioned by the reviewer I shall not I trust be accused of anything rude or uncivil, in asking whether this gentleman has served any considerable portion of his time in the East or West Indies, if so, I have only to observe that he differs very widely in opinion from others similarly situated. Hear the opinion of one of the survivors of the mortality at St. Domingo,—one who subsequently served through the Peninsular war with the elevated rank of a Deputy Inspector of Hospitals, one who, a second time, served in the West Indies, as Chief of the Medical Staff in the Windward and Leeward Islands, and who now lives respected and esteemed by every man who knows him. "I ought," says this voice from St. Domingo, "to have thanked you before now for the little brochure you have sent me, in every word of which I thoroughly agree, and only wish that your arguments may make a suitable impression upon the authorities who have power to give them effect. Your quotation from Mr. John Bell is singularly apposite and good. The most experienced campaigner could not have written better." Hear again the opinion of another gentleman who retired as President of the Medical Board after seven and thirty years' experience in various parts of India;—under all circumstances and situations of inter-tropical service; in charge of large general hospitals at fixed stations; in field hospitals on actual service; in regimental hospitals moving over various countries, and through different climates; amongst Europeans as well as Natives; and among men, women and children; "I am very much obliged to you for your kind letter and enclosures, I sincerely hope you may be successful, and I think it very probable you will, as the importance of what you recommend must be obvious to every man who thinks at all, and particularly those who have any knowledge of soldiering, or the important duties devolving upon medical men on Foreign service. You have already done so much to benefit and improve the studies of Military Medical Officers that nothing is left but to follow up your plans; which, if adopted in London and Dublin, by the nomination of competent Professors, there cannot be a doubt that the public will be great gainers."

Both the gentlemen above alluded to have written much, and have written well, on the diseases of the troops, and on military hygiene, and their cordial approbation of my views would seem to shew, that they concur in opinion with another talented and experienced Army Surgeon, who, with reference to the very article on which I am now commenting, observed that "the reviewer might as well propose to supplant the Commissariat of an Army by Dr. Kitchener's work on Cookery, as to supply the purposes of so great a School by a book." But it were endless to multiply such quotations, suffice it to say that I am now in possession of six-and-forty letters to the same effect—all these letters, be it observed, wholly unsolicited. One from the Director-General; seven from Inspectors of Military Hospitals, or of Naval Hospitals and Fleets, eighteen from gentlemen holding the rank of Staff, Regimental, or Naval Surgeons, and the others from Professors in English Universities, from private practitioners, or from General and Field Officers in the Army—the last a class of men whose evidence I am inclined to rate very highly in a question of this kind. A medical man does not, I believe, consider it necessary to acknowledge every pamphlet which may be laid upon his table, and when so many gentlemen of talent and experience have stepped forward to volunteer their opinion in support of my proposal, I may well sit at ease under the strictures of my reviewer.

I conclude by observing that it is from respect to the established reputation of your Journal, from respect to your own character, personal and professional, and from respect to your position as a Naval Surgeon, that I have been mainly induced to enter into this detail. Believe me, Sir, that in what I have now written I am much more solicitous about the promotion of a great public object, than about my personal vindication, or my private interest. I have now been employed for nearly forty years in the study, in the practice, and in the teaching of Military Surgery. I have submitted to the Government, from an imperious sense of duty, my sentiments on this important question, *valeant quantum valere debent.* Gratified by the approbation of my seniors and cotemporaries, both Military and Medical; strong in the rectitude of my purpose; and feeling, personally, but little interested in the result, I shall not again be easily drawn from that quietude which best befits my habits, my present state of health, and my time of life. I remain, Sir,

Your very faithful and obedient Servant,

GEO. BALLINGALL.

---

WYKE HOUSE ASYLUM FOR NERVOUS INVALIDS OF THE UPPER AND MIDDLE CLASSES.

It is only among the Poor that the benevolent views of a more advanced Science, in the treatment of the Insane, can be said to be reduced to practice. Among the middle and upper Classes they are still unapplied, at least to any satisfactory extent. Persons of moderate means, or who are not too rich to disregard expense, are debarred the advantages of the well-organized Pauper Asylum, and are too often made over in consequence to the care of men destitute of any notions of medical or psychological science. We have laid aside the means of force, but in how many forms do neglect, harshness, and abuse still prevail! We have ceased to regard these poor creatures as brutes ; but have we yet begun to treat them as men ? The alienated invalid is but too often abandoned to the care of a keeper, without employment, recreation, or personal consideration. He is cast out, because he is stricken ; and hence his restoration is rendered more uncertain, and often hopeless.

It is to meet this want that Dr. Costello, long the pupil and friend of the illustrious Gall, has opened Wyke House, Osterley Park, not with the pretension of forming a model Establishment, but to aim at least at making better arrangements for these classes of patients than have hitherto been brought within their reach.

In order to carry out a plan based on the accommodation of numbers, without which the advantages of a judicious classification are unattainable, the first desideratum was an extensive range of buildings in a favourable locality, with pure air and water, and a sufficient extent of land to afford facilities for exercise, varied occupations, and quiet seclusion. These conditions, indispensable to a well-combined plan of general treatment, are united in Wyke House, almost to a degree unequalled by any Establishment in the kingdom.

The House is situated on Sion Hill, one of the most elevated and salubrious spots in the vicinity of London, from which it is distant 7½ miles. The buildings consist of a large mansion, fronting to a woody lawn, with two wings extending from it, and enclosing a spacious court, 200 feet long. The facades, front and rear, extend 270 feet. The flower and walled gardens, and ornamental grounds, cover an area of nine acres, and, inclusive of the farm lands interspersed with finely-timbered groves, the range extends over 30 acres. The command of prospect is extensive : Harrow, Hampstead, Richmond, and the Surrey Hills, are in the distance : while Ealing, Hanwell, and the winding valleys of the Brent and Thames, form the fore-ground. It is thus the centre of some of the most attractive drives in Middlesex, viz., Osterley, Sion, Ealing, Kew and Richmond Parks, Isleworth, Twickenham, Hampton Court, Bushy, and Sunbury.

Within wholesome bounds, there are still many enjoyments, in which, either for cure or comfort, the nervous invalid ought to be made to share. This being a leading principle, the Establishment for all who are capable of social relaxation forms but one family, of which the Director and his lady are the heads. Influences opposed to the morbid fancies of the patient are thus brought into activity, at table, and in the evening circle, by music, conversation, books, and the numberless offices of kindness and attention of an uninterrupted intercourse. By such means he is gradually strengthened to shake off his wayward imaginings, and to feel that he is not in a prison, but in a home.

The medical and moral treatment embraces all the modern improvements—baths, douches, padded rooms, chair and carriage airings, &c. The out and in-door occupations and recreations comprise the culture of plants and flowers, the management of hot and green houses, of domestic animals, the farm yard, and horsemanship ; billiards, tennis-court, skittles. The airing-grounds are large and dry ; and there are spacious rooms for exercise in wet weather.

*For particulars, application to be made at No.* 10, *Golden Square, in London ; or at Wyke House, Sion Hill, Middlesex.*

### Circular addressed to the Medical Officers of the East India Company's Service, by the Medical Board at Calcutta. Dated 20th October, 1818.

*To the Editor of the Medico-Chirurgical Review.*

Oriental Club, 31st May.

Sir,—In your number for April last, you published an interesting but visionary letter from the late Mr. John Bell, to the late Earl Spencer, in relation to the means of promoting Military Medical Science. Will you be so kind as to find a place in your forthcoming number for the accompanying appeal to the Medical Officers of the East India Company's Service, by the Medical Board at Calcutta, on a similar subject. The "Circular," which was obviously much called for, is dictated by sound sense and great practical experience.

I am, Sir, &c.

Orientalis.

Sir,—In conformity with the orders of Government the Medical Board have directed me to submit to you the following considerations, relative to a proposed plan for the collection, and accumulation, of professional information, relative to this country, and its more extensive diffusion, amongst the medical part of the service under this Presidency.

It has been a frequent subject of regret to the medical world, that, wide and rich as is the field in India, for the cultivation of physical science, and the study of pathology, the labour hitherto employed in it, even in comparison with what has been bestowed on far less promising spots, has been negligent and irregular, and the product presented to the public, scanty, and in the extreme unprofitable. Whilst in almost every other portion of the civilised world, scientific objects have been pursued with energy and success, and every successive year has largely added to the previous general stock of medical knowledge; India has scarcely given birth to a single important discovery, or publication of acknowledged merit, on professional subjects, since the first establishment of tl e British interests in this quarter.

If we search for the causes which have given rise to this strange anomaly, and placed these Settlements apparently so far behind the other scions from the parent Country in point of professional zeal and intelligence: we shall not find that it can be fairly attributed to the faulty constitution of our Medical Establishment; or to the apathy and incapacity of those belonging to it. The individuals composing this respectable class, have ever been remarkable for the diligent and able performance of their duties. They would seem to be, by previous regular education, and by subsequent large acquaintance with the diseases usually prevalent in hot climates, peculiarly qualified to impart useful information to their less experienced brethren.

The evil, then, is to be traced to another source: and will be readily discovered in the total want of any eligible means of communication between them and the profession at large. Removed as they are, to an immense distance, from Europe, they cannot venture on the risk, or undertake the superintendence of printing a large work; and, in this remote spot, they have not, as in London, and other great cities, periodical journals exclusively devoted to professional subjects; and affording facilities for publishing, without expense or trouble, such short essays, or individual facts, as they might deem worthy of general notice.

These peculiarities of exclusion, to which the Medical Staff of this country are necessarily exposed, have been productive of very serious disadvantages. They have affected equally the reputation of the individual, and the character of the profession at large. The fruits of each person's experience have, unavoidably, been confined within the narrow sphere of his individual knowledge, or uselessly deposited amongst the records in office, and thus, whilst the medical world has been deprived of the principal end and use of past observations,—that of serving as a guide to practitioners for the future, the younger branches of the profession have been shut out from benefiting by the fund of knowledge acquired by their seniors.

Strongly impressed with the truth of these remarks, and fully convinced of the expediency of removing the injurious barrier, at present existing between the medical observer and his brethren, Government has lately resolved upon collecting and printing at the public expense, all communications of interesting facts and particulars connected with the diseases of this country, and the modes of treatment best adapted to their removal. Instructions have accordingly been issued to the Medical Board, to make the substance of this resolution generally known throughout the Medical Department of the Service, and to carry the subordinate arrangements for the success of the plan depending on it into early effect. It is in compliance with these instructions, that I have now the honor to address you.

After mature reflection, it has appeared to the Board, that the most ready and effectual means of fulfilling the liberal intentions of the Most Noble the Governor-General in Council, is to hold out an invitation to all officers borne on the medical list, to prepare and transmit to this office, such papers, whether comprehending a general description of any disease, or set of appearances, or confined to the relation of single cases or remarkable facts, as they may be willing to lay before the public.

The nature of the projected compilation, and of the materials of which it is desirable that it should be composed, may be readily conjectured from the foregoing remarks. It will thence naturally be supposed that the greater portion of it will be devoted to subjects of a strictly professional nature. It is by no means the wish of the Board to exclude from it essays, or single observations on chemistry and its dependent arts, natural history, botany, or others of the sister sciences. One of the most gratifying effects indeed contemplated by the Board from the successful operation of the scheme under consideration, would be the direction of a greater share of attention than has been hitherto given to these very interesting departments of science; the investigation of which, besides the important uses to be derived from it, affords a copious source of rational enjoyment to an inquisitive mind.

On the choice of subjects for publication, the medical observer must therefore, in a great measure, be left to himself, and each individual will, of course, on this point, follow the bent of his own inclination: the Board are convinced that he can never be at a loss for fit points of inquiry. The true nature and treatment of fevers, bowel complaints, and the other great classes of disorders common to India, with other regions, are yet very imperfectly understood. And their investigation, while it lies peculiarly open to practitioners in this country, would be hailed by their brethren in Europe, as opening a path that must lead to conclusions important and interesting to all. The various species of cutaneous diseases, elephantiasis and other maladies almost peculiar to warm countries, present a wide and nearly untrodden field to the curiosity of future investigators, whilst much room for experimental research still remains in ascertaining the real powers of such articles of the materia medica of the natives as seem to possess virtues peculiar to themselves, or as might be occasionally substituted with advantage for medicines imported from Europe.

So soon as a body of manuscript, sufficient to form a sizeable volume shall have been collected, the Board will proceed to digest and prepare it for the press. The Board understand it to be the present intention of Government, that copies of each successive volume, shall be distributed to every medical officer throughout the country. They wish it to be clearly understood, that of the papers transmitted for their revision, it will be optional with the individuals making the several communications, to determine whether their names shall or shall not be made known to the public. They do not anticipate many instances in which it will be necessary for them to exercise the rejecting power placed in their hands; but should it ever happen that they find themselves obliged to perform this painful duty, the papers meeting their disapproval will, whenever required, be returned to their authors.

Having thus alluded to the materials of which the proposed work is to be framed, it seems proper to say a few words on the part to be taken by the Board in their compilation and preparation for the press, it will appear evident that some power of alteration as well as selection must pe placed in their hands; but this power, it is both their wish and determination to use as sparingly as possible. From the communications received, they will set apart and arrange for publication, without alteration, all papers appearing to contain important doctrinal views and interesting practical details and observations; this will generally be done without minute reference to judiciousnsss of manner, or peculiar niceties of style, as occasion may seem to require; they will preface each class of essays on any particular disorder, with such remarks as their own longer experience, or various returns from the different establishments under their control may point out to them as bearing upon the subjects under discussion. In addition to these prefatory remarks, short notes may now and then be interspersed at the foot of the page elucidatory of the text, and communicating such information as may prove generally interesting to the profession, or such recent medical intelligence, as although known to their brethren in Europe, may, from distance and the difficulty of procuring English publications at remote stations, still have failed to reach the great body of the medical staff in this country. To each separate volume the Board may likewise prefix an introductory discourse, comprehending a historical sketch of the health of the various corps and departments of the army, and of the civil establishments under their superintendence, during the past year, and illustrative of the causes and effects of any sudden or marked changes; any increase or diminution of sickness, that may have occurred during the period under review. The whole to be followed by a series of tables or returns, showing the strength of each corps; the number of its sick and casualities, and the complaints most prevalent amidst it, and affording to the profession in general data, on which to form accurate conclusions regarding the nature and fatality of the diseases usually encountered in Indian climates.

The Board need hardly say, that in a work, one main object of which is to excite a free spirit of enquiry and discussion, they cannot consider that it falls within their province to censure harshly any portions of individual essays, that may, in their judgment, contain assumptions erroneous in theory or practice, to constitute themselves absolute judges of the merits of questions at issue; or to assume authority, to dictate precise rules, or lay down positive laws, for the guidance of the Medical Staff in the treatment of diseases. They will, in all cases, consider it to be their duty to refrain from taking part in professional disputes, and from betraying such a bias to particular theories or opinions, as might tend to restrain those under their authority, from the open expression of their sentiments; and, if they ever interfere in the points under discussion it will be from the more gratifying motive of holding up to general approbation, instances of rare ingenuity, or of laudable industry.

The great and numerous advantages to be gained by the prosperous issue of the undertaking now on foot, are so clear and evident, that the Board feel perswaded they need make use of no further arguments to secure to it your concurrence and hearty co-operation. Its immediate effects, it is hoped, will be to excite a spirit of honourable emulation, that may call forth the talent and energy which have till now lain nearly dormant: to give a strong impulse of activity, and to communicate to all parts of the Service a feeling of consent and mutual respect, and an ardent desire for the improvement of professional pursuits. Looking a little further, we may venture to predict, that ere the lapse of many years, a large mass of valuable information will, by its means, be placed before the public, and this country be thus relieved from the reproach under which she has long lain, of having failed to contribute her part to the advancement of science, and the lessening of disease and human misery.

Such communications as you may please to make in furtherance of the foregoing statement you will have the goodness to address, in the usual form, to this office.

I have the honor to be,

Sir, &c. &c.

SECRETARY, MED. BOARD.

